# Remarkable Malformation

**Published:** 1868-03

**Authors:** W. S. Carter

**Affiliations:** Pittsboro, Ind.


### ARTICLE V.

### *Remarkable Malformation.*

By W. S. Carter, M.D., Pittsboro, Ind.

Mrs. W. was delivered, after an easy labor at full term, of a living male child. The infant was perfectly quiet for a few minutes after its birth, and then spasmodic respiratory efforts were made. Thinking the throat might be obstructed by mucus, I endeavored to introduce my fingers to remove it. The fingers passed readily between the lips, but to my astonishment I could get it no further than the gums, which both by sight and touch I found firmly united.

As it was necessary to act promptly, I immediately, with the assistance of my partner, Dr. Tilford, divided the tissue uniting the gums. This appeared to be about as thick as the gums, and cartilaginous, extending as far back on either side as the angle of the jaw. Notwithstanding this free division, which enabled the child to breathe with more facility, the jaws were immovable.

After letting the patient rest a few hours, Dr Sellers, of Brownsburg, visited the patient with me; and it was decided to use some force to separate the jaws, and make a further careful exploration. This exploration showed us a tough membrane, one-eight of an inch in thickness, passing from the palate bone above, and inserted into the lower gum. Upon the division of this, and the use of some little force, the jaws were separated.

In two weeks the gums had healed, the child took nourishment readily, and was doing well.

Other malformations also existed in this case, viz., the fingers and toes were webbed, and the ears were in rather a rudimentary condition—the integument passing from the head over the anterior surface of the upper third of each of these.

When the mother was about three months pregnant, her son, about six years of age, had a severe convulsion, the jaws being spasmodically closed. She was alone at the time, and her terror was excessive; and, indeed, since then, during all the remaining months of her pregnancy, she states the frightful scene has scarcely ever been absent from her mind.—*Western Journal of Medicine.*

